# EHMTI-0263. WHO step scheme into combination with a therapeutical local anesthesia (TLA), physiotherapy, TENs, phytotherapy, acupressure, acupuncture for chronically therapy resistant trigeminal neuralgia after Trang

**DOI:** 10.1186/1129-2377-15-S1-C47

**Published:** 2014-09-18

**Authors:** T Nguyen, A Argyrakis, TT Nguyen, H Eckel, W Vogelsberger

**Affiliations:** 1 Pain Therapy, Praxis, Göttingen, Germany; 2 Neurologist, practice, Bad Karlshafen, Germany; 3 Childrens Clinic, University clinic, Bad Karlshafen, Germany; 4 former boss doctor, Radiology, Göttingen, Germany; 5 Specialist Anaesthesiology, former boss doctor, Freiburg, Germany

## Introduction

The Trigeminal neuralgia is associated with immense pains besides the destruction pains at myocardial infarction whose intensity makes the life to many of the patients concerned the hell, frequently leads with patients to suicide.

## Objective

The extremely therapy resistant pains got more tolerable, used for the preservation of the success and further recovery at continuation of the therapy in the four to paying attention-week treatment distance. With all patients the quality of life improved significantly because of the treatment and the pain diminution resulting from it.

## Methods

The therapy after mentioned WHO step scheme after Trang with PDA C2 - C4, Ganglion pterygopalatinum blockade, physiotherapy, psychotherapy, TENS, Phytotherapy, cupping, acupressure, acupuncture, could the chronically therapy resistant Trigeminal neuralgia remove the pat. at over 70% completely, obtain a recovery at the left to 70% of the original complaints, in which at first the started therapy was used accompanyingly furthermore until a longer pain reduction with Carbamazepin or Neurontin.

## Results

This therapy has already helped Trigeminal neuralgia many patients therapy more resistantly with before. None of the patients given to therapy had to suffer from pains before unbearable for his furthermore so that the modified WHO step scheme after Trang means a new breakthrough in the treatment of the Trigeminal neuralgia.

## Conclusion

The therapy techniques are demonstrated in support of everyone understandably here.

No conflict of interest.

